# Not putting the cart before the horse: the complex social and ethical terrain of prenatal exome sequencing

**DOI:** 10.1038/s41431-022-01225-4

**Published:** 2022-11-07

**Authors:** Felicity Boardman, Ruth Horn

**Affiliations:** 1 Warwick Medical School, Gibbet Hill Road, Coventry, CV4 7AL UK; 2 The Ethox Centre, Nuffield Department of Population Health, University of Oxford, Oxford, UK; 3 Ethics in Medicine, Faculty of Medicine, University of Augsburg, Augsburg, Germany

**Keywords:** Genetic testing, Pregnancy outcome

Prenatal exome sequencing (PES) for the diagnosis of foetuses ‘with a likely monogenic malformation disorder’ has been available through NHS care in the UK since 2020 [1]. Foetuses undergoing PES typically have anomalies that have been identified through sonographic imagining, and in these instances, a molecular diagnosis is sought to inform management of the pregnancy and/or neonatal care. Indeed, across the globe, the benefits of PES when used in addition to chromosomal microarray analysis (CMA), have been widely emphasised. These benefits not only include diagnostic yields, but also the facilitation of informed decision-making regarding pregnancy dis/continuation, early diagnosis, as well as initiation of pre-symptomatic treatment [2, 3]. Despite these important potential benefits, however, the use of genome-wide approaches to prenatal testing also requires careful consideration given the number of ethical issues that have yet to be adequately explored [4].

Daum et al.’s recent paper, makes the case for expanding the use of PES to foetuses where no anomalies have been previously identified- in other words, suggesting that PES could be usefully employed as a first-tier screening test. The authors claim that PES, even in pregnancies without a specific indication, ‘includes all diagnostic yields of CMA and provides additional findings in a considerable fraction of seemingly healthy foetuses’ (p.11). Their argument for the movement of PES out of the realm of targeted diagnostics into that of screening is therefore based, primarily, on diagnostic yields, and a concern that CMA alone may provide ‘false reassurance’.

Their paper reports on PES conducted on 482 ‘structurally normal’ foetuses between 2017 and 2022 in Israel. In order to determine which results to disclose to parents, the team used the American College of Medical Genetics and Genomics (ACMG)’s list of pathogenic, and likely pathogenic variants, as well as the ACMG’s list of secondary findings [5]. These lists were then further syphoned down by including only those relating to ‘moderate’ or ‘severe’ conditions, as determined by Lazarin et al’s (2014) taxonomy [6]. Reporting of ‘secondary findings’ were restricted to those with childhood onset, but, importantly, still including susceptibility genes. Using this system, the study identified four foetuses with pathogenic, or likely pathogenic, variants relating to a ‘moderate’ or ‘severe disease’ (all of which were subsequently terminated) and two foetuses with childhood onset secondary findings (both continued to term).

Although the authors emphasise the benefits of this approach in facilitating informed decision-making in pregnancy (in the absence of pre-conception carrier screening), we believe that there are a number of ethical issues deserving of attention before ES could be seriously considered as a routine form of prenatal screening of ‘structurally normal’ foetuses.

Firstly, the approach used by the authors to determine which variants should be reported back to would-be parents, we believe, requires more thought. Several authors, including Van Rooijk et al. (2020) have argued that the label ‘known pathogenic’ should be applied to genetic variants only very sparingly, given that many that fall into this category do not have a clinical impact in every instance [7]. Whilst van Rooijk et al.’s study was carried out in an older population, the findings may nevertheless be important to consider in the prenatal context where there is limited opportunity to assess the impact of a variant on phenotype. In addition, many variants identifiable through PES (including two found in this study- in SPRED1 and FGFR3) are associated with conditions with wide spectrums of presentation (Legius syndrome and Muenke syndrome respectively), further reducing the ability of PES findings to accurately predict the future clinical impact. Even in instances where pathogenicity, penetrance and expression of a variant are well characterised, the boundaries between ‘mild’, ‘moderate’ and ‘severe’ conditions (which were so pivotal to the authors’ disclosure policy) are not clear cut. In recent years, there has been increasing acknowledgement of the difficulties of relying on taxonomic systems for the classification of condition severity, and of the frequent mismatch between clinical interpretations of life with genetic disease and the perspectives of those who actually live with them [8].

Secondly, and related to the above point, the inherent difficulties with defining terms such as ‘pathogenicity’ and ‘severity’ mean that the likelihood for uncertain or equivocal results being returned through PES is extremely high. This prospect is especially concerning given the potential impact of the results on would-be parents, and their likely influence on decisions around dis/continuation of a pregnancy. Previous research demonstrates the profound psychosocial distress that can follow uncertain prenatal results [9], with cultural, religious and social factors being drawn on to aid interpretation of a future life affected by a (frequently unfamiliar) disability. However, filling the void created by an absence of biomedical certainty with social and cultural interpretations of disability can, in itself, be problematic. Research has demonstrated that poor public knowledge and understanding of both genetic conditions and disability can hamper the ability to accurately imagine future lives affected by genetic conditions [10]. Indeed, whilst Daum et al. have emphasised the ‘false reassurance’ associated with use of CMA without PES, we argue that its inclusion could conversely introduce new uncertainties, decision conflict and distress amongst prospective parents, as has been demonstrated in other prenatal contexts where equivocal results have been identified [11]. In light of this, achieving valid consent for ES, will not only require counselling about the ‘limited phenotypic delineation of the foetus’ in variants with variable expression and incomplete penetrance (Daum et al., p. 4), but also discussion of the individual meaning of severity and disability for the woman/couple, the (uncertain and variable) meaning of pathogenicity in advanced genomic testing, and findings that go beyond the initially defined list of genetic conditions [12].

Finally, further discussion is needed around how we define ‘benefit’ in the context of ES in pregnancies without indication. Benefit should not only be evaluated from a clinical perspective, but also from women’s perspectives, public health perspectives, and from a health economic perspective. This type of analysis requires in-depth empirical investigation of women’s experiences as well as solid economic evaluation to avoid unjustified strain on already overloaded healthcare systems. The latter requires taking account of the costs of data analysis, interpretation and curation as well as of clinical time required to discuss findings with patients [13].

In summary, it is our belief that it is important not to overemphasise the potential benefits of PES and understate some of its inherent uncertainties and potential harms. The uncertainties and limited diagnostic performance of ES should be acknowledged not only in the context of ‘mild’ to ‘moderate’ foetal disease, as suggested by the authors (p.10), but also with regard to ‘pathogenicity’ and ‘severe disease’- the definitions of which remain both fraught and contested. It is our view that further social and ethical research is needed to explore the wide range of perspectives (including those of women and disabled people themselves) that can be meaningfully brought to bear on the uncertainties and complexity that currently surround PES results. It is only through such analyses that the wide-ranging social and ethical implications of PES can be fully understood, and before PES could ever be seriously considered as a first-tier screening test.
